# A new digital model for the Italian Integrated Home Care: strengths, barriers, and future implications

**DOI:** 10.3389/fpubh.2023.1292442

**Published:** 2023-11-14

**Authors:** Fidelia Cascini, Andrea Gentili, Andriy Melnyk, Flavia Beccia, Francesco Andrea Causio, Vincenzo Solimene, Serena Battilomo, Simona Paone, Alice Borghini, Michelangelo Bartolo, Emilio Chiarolla, Walter Ricciardi

**Affiliations:** ^1^Section of Hygiene, Department of Life Sciences and Public Health, Università Cattolica del Sacro Cuore, Rome, Italy; ^2^General Directorate of Health Information System and Statistics, Ministero della Salute, Rome, Italy; ^3^Italian National Agency for Regional Healthcare Services (Agenas), Rome, Italy; ^4^Telemedicine Department, San Giovanni Addolorata Hospital, Rome, Italy

**Keywords:** digital health, teleassistance, telemonitoring, televisit, telehealth, home assistance

## Introduction

Telemedicine is the remote delivery of healthcare services using information and communication technologies for the exchange clinical data for diagnosis, treatment and prevention of diseases (1). Telemedicine has historically been used to provide healthcare to rural or underserved areas. Still, in recent years it has grown in popularity as a way to improve access to healthcare, increase care quality level and, in some cases cost reduction was proved (2). However, the regulatory landscape for telemedicine varies across countries and regions, with some having more developed and supportive frameworks than others (3). The use of telemedicine in the National Health Service (NHS) in Italy peaked during the COVID-19 pandemic, which resulted in the development of the Italian National Recovery and Resilience Plan (“*Piano Nazionale di Ripresa e Resilienza”*, PNRR) (4). The PNRR is part of the Next Generation EU (NGEU) programme, namely the €750 billion package that the European Union negotiated in response to the pandemic crisis with the goal of strengthening local prevention and health services, modernizing and digitalizing the health system and ensuring equal access to care.

Those objectives will be achieved through:

- the creation of Community Health Houses (“Case delle Comunità”) and Community Hospitals (“Ospedali di comunità”) for proximity healthcare;
- new Territorial Coordination Centers (“*Centrale Operativa Territoriale”*, COT) for remote healthcare; and
- technological and digital upgrading including diagnostic imaging machinery.

The new healthcare delivery model is based on patient access and continuity of care in a multi-stakeholder process. The patient's home is the first and main place of health delivery, and COT will act as a *trait-d'union* between healthcare services and professionals, playing the role of transitional care following patient through different settings of care. The implementation of telemedicine is pivotal: within Mission 6 of the PNRR (“Health”), the component “Territorial assistance and telemedicine” has the goal of reaching out to at least 10% of the older adult population (over 65 years of age) to tackle the issue of patients with chronic conditions lost to follow-up and who only access the healthcare system when developing acute complications. In order to do so, it is planned to assist through telemedicine at least 200 000 people by the end of 2025. The main objective of the National Portal for Telemedicine is to establish a fundamental level of interoperability that ensures common standards for telemedicine services developed by the regions, enhancing what is already available in the landscape of local contexts while complementing or completing the portfolio of services. In particular, it aims to connect all healthcare providers and patients in Italy, and the dissemination of electronic prescriptions and Electronic Health Records (EHRs) (5).

This paper aims to describe the technical, structural, and operational characteristics of the new Italian Integrated Home Care (IHC) digital model, as described in the most recently operational manual published by the Ministry of Health. These features will be discussed and analyzed in order to identify strengths, weaknesses, and future implications, as well as to assess how the proposed model fits into the European context, and possibly comparing it to other highly advanced international ones.

### Description of technical, structural features

**Figure 1:**
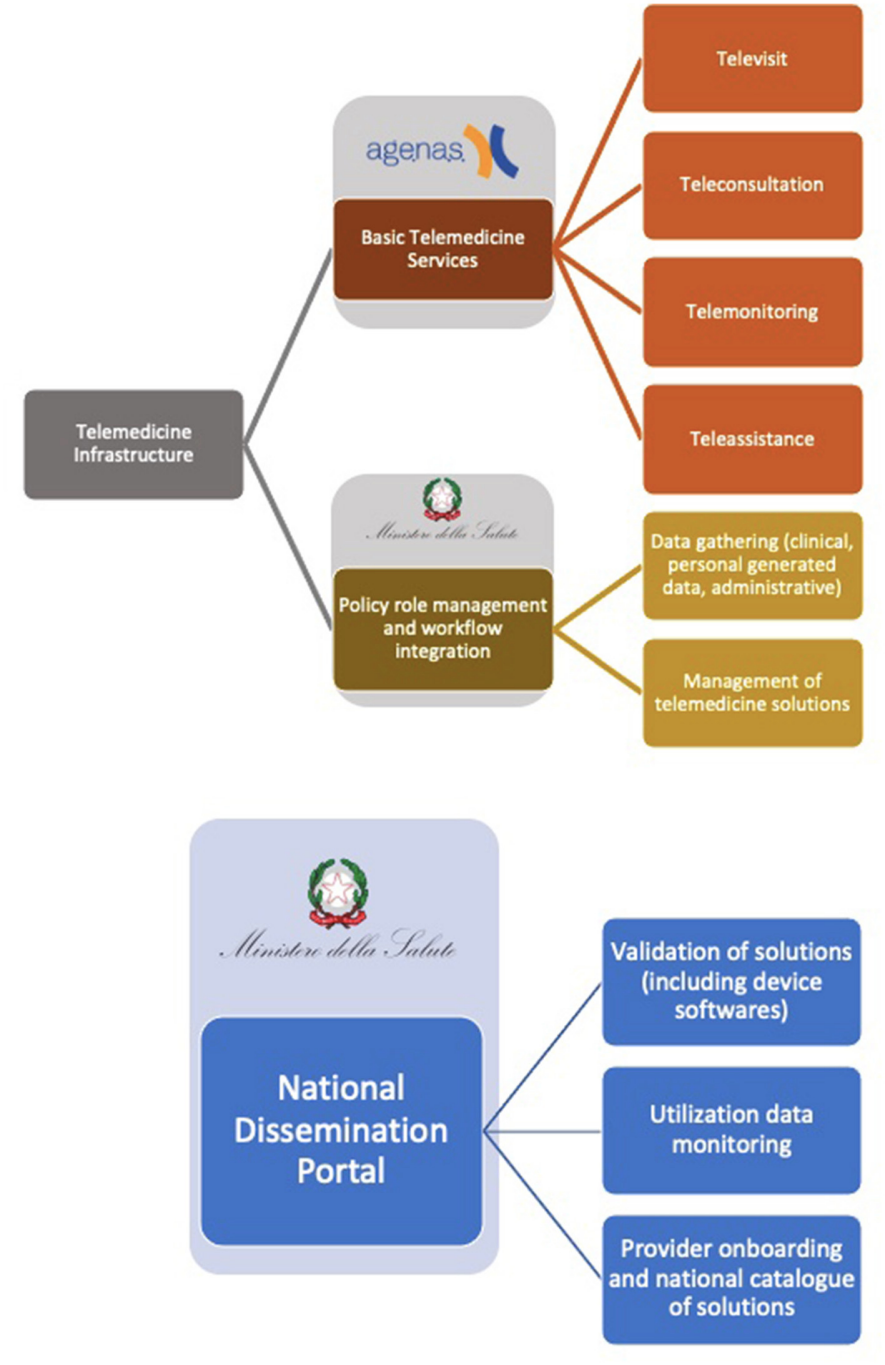
Description of the main services provided by each infrastructure in the national telemedicine model and the entities responsible for their development.

The National Digital Health Agency (“*Agenzia Nazionale per la Sanità Digitale*”) is charged with ensuring the provision of telemedicine services through the cloud-based Network Time Protocol. The Network Time Protocol is a national infrastructure which contains the “Enabling Services” (data gathering, management of telemedicine solutions, policy role management and workflow solutions) and all the connected regional infrastructures which provide “Basic Telemedicine Services” (healthcare professionals' teleconsultation, telemonitoring, teleassistance, patient's teleconsultation known as “*televisita*”) (6). Communication between different services, infrastructures and local health authorities (Aziende Sanitarie Locali—ASL) is guaranteed by a modular interoperability layer in which various pathways ensure data standards and quality, smoothing data governance. Finally, a national telemedicine dissemination portal (“*Portale Nazionale per la Diffusione della Telemedicina*”), controlled directly by the Ministry of Health will contain data on all telemedicine solutions provided (Figure 1).

### Operational and organizational aspects of the new digital home care guidance

In Table 1, four main use cases (patients' teleconsultation, healthcare professional teleconsultation, telemonitoring, teleassistance) will be examined and structured following the newly published ministerial guidance for the implementation of digital home care (7, 8). In each use case, it will go through the prescriber, the activation and provision service, and the technical support and shared information.

**Table 1:** Organizational aspects of the 4 main use cases (patients' teleconsultation, healthcare professional teleconsultation, telemonitoring, teleassistance).

	**Patients' teleconsultation (“*televisita*”)**	**Healthcare professionals' teleconsultation**	**Telemonitoring**	**Teleassistance**
Description	Patient Teleconsultation refers to interactions between a clinician and a patient to provide diagnostic or therapeutic advice through electronic means. According to Italian legislation^*a*^, patient teleconsultation is only allowed to monitor patients who have already been diagnosed with a condition/disease during a in-person appointment. A teleconsultation could have numerous goals, such as: • Follow-up of a previously known condition. • Adjusting or changing therapy. • Taking a medical history and clinical evaluation for the prescription of medicines or diagnostic exams. • Sharing with other relevant professional updates on the patient's medical journey/diagnostic pathway.	Healthcare Professionals' Teleconsultation is a medical procedure in which the healthcare professional interacts remotely with doctors and colleagues to discuss the patient's clinical case. During this process, it is possible to share all the relevant data, reports, images, audio-visual material, regarding the specific case.	Telemonitoring enables medical professionals to gather vital and clinical parameters remotely and patients to transmit them via body sensors that interact with the patient (biometric technologies) The remote technological set is personalized according to the physician's specifications. The patient must be constantly connected to the software that collects data from the body sensors. The clinical data is combined with other relevant data, and then shared with all the various stakeholders involved in the patient's care process.	Teleassistance can be considered as a hybrid between telehealth and telemonitoring. It is a professional act pertaining to the related health profession and is based on the remote interaction between the professional and patient/caregiver by means of a video call, which can, if necessary, be added the sharing of data, reports or images. The professional who performs the telehealth activity can also use suitable apps to administer questionnaires, share images or video tutorials on specific tasks. The purpose is to facilitate the proper performance of care activities, which can be mostly delivered at home.
Prescriber	General practitioners, health authority doctors, and medical specialists could request a teleconsultation through a prescription. This service may be provided to all patients (even those with non-complex management) and it does not require a multidisciplinary evaluation or individualized care plan.	In the IHC setting, general practitioner, pediatrician, health authority doctors, health professionals or specialist prescribe a teleconsultation. This service could also be extended to patients with non-complex management, and it does not require a multidisciplinary evaluation or individualized care plan.	In the IHC setting, telemonitoring may be deemed necessary by a general practitioner, pediatrician, health authority doctors or specialist. Telemonitoring always requires a clinical pathway that has the following defined: number of eligible patients, available telemonitoring tools, minimum set of monitored parameters, threshold values, selection of data to be reported in the clinical documentation, alarm management pathway, as well as professionals for the management of interventions (call of the referring nurse or physician, home access of the healthcare professional, *televisita*—i.e. patient's teleconsultation, emergency room activation).	General practitioners, health authority doctors, and medical specialists could request teleassistance through prescription. This service may be provided to all patients (even those with non-complex management) and does not require a multidisciplinary evaluation or individualized care plan.
Activation and provision	The responsibility for activating the process lies with the COT, general practitioners, pediatricians, and specialists. The teleconsultation will be provided by healthcare professionals, which will be able to annotate the outcome of the consultation in the EHR.	As this type of teleconsultation consists in an interaction between healthcare professionals, the activation may be direct (even through shared agendas) when communications pathways are pre-established. Otherwise, the activation can be done indirectly through the COT, or the operations center of the IHC setting, that is the control room of the IHC service.	In the case of patients already under the care of an IHC service, the activator is the control room, if present. In the case of patients moving between different settings, where the coordination of different specialists and competences is needed, the activator is the COT. The Center for Telemedicine Services following: Telemonitoring service (such as access to the portal, setting, threshold values, etc.), technical assistance and second level help desk service (technical support) in case of need (through channels, times and days defined).	The activators of the teleassistance services are: the aforementioned health professionals, the COT, and the IHC Operations Center (where present). Activation of the service should be planned appropriately based on agendas, observations shared with the health professionals involved, and managed directly by them or the IHC Operations Center where present or COT. The providers, however, are the health professionals.
Technical support and information shared	There is minimum basic equipment required to support the teleconsultation. During and before the visit, it is important to always ensure the possibility to share real time clinical information, data, medical reports, images, and video. Patients' information must be available in the EHR where healthcare professionals may retrieve or store data and information. When patients do not meet the requirements of both clinical and technical compliance to perform the teleconsultation, the medical appointment must be done in-person.	In order to initiate a video call, basic technological equipment is necessary. Tools facilitating the consultation and sharing relevant clinical documentation (reports, images, etc…) are deemed essential. Patients' information must be accessible to the IHC service.	Telemonitoring is feasible thanks to specific set of certified technological devices, connected to a central portal to receive and properly store the obtained data. Portal and data access must be allowed by the physician that prescribed the telemonitoring and by the equipment involved in the clinical pathway. The relevant information contained in the portal and useful to taking care of the patient at home are: - Data periodically detected by the telemonitoring system. To guarantee patient's continuity of care, this data may be reported in the home care folder, with an automatic import. - Periodic evaluation of the telemonitoring system, performed by the general practitioner (or pediatrician) that has the management of the case.	The instrumentation provided for teleassistance consists of devices for recording, data and image storage, software for data and image exchange, video and vital parameters, medical devices and sensing sensors. Additional possibilities are provided by linking and consultation of data or information detected during the service itself. The information present in the teleassistance portal is: • data periodically surveyed by the professional. • data periodically self-detected and entered by the caregiver. • documents: reports and notes of the various visits/interventions performed, assessments by the health professionals, or by the physician in charge of the case and of the actions taken (diet update, drug therapy update or other, based on changes in the health status due to the disease).

IHC, Integrated Home Care. ^*a*^Piano nazionale di ripresa e resilienza Missione 6: Salute Componente 1 (M6C1): Reti di prossimita', strutture e telemedicina per l'assistenza sanitaria territoriale. Investimento 1.2.3 La telemedicina a supporto dei pazienti nell'assistenza sanitaria territoriale. Linee guida per i Servizi di telemedicina. Requisiti funzionali e livelli di servizio (Allegato A).

## Discussion

### Strengths

Similar to many other European countries, Italy is expected a strong decline in population (especially in the rural areas) within the next 10 years (9). One of the most immediate and pronounced effects of a declining population is an aging demographic. As the proportion of older adult individuals increases, there will be a strain on healthcare systems and pensions. As rural areas depopulate, they may face challenges related to maintaining infrastructure, schools, and healthcare services.

Several countries have implemented telemedicine as an effort to improve healthcare for residents of rural or underserved areas, where healthcare facilities and specialists may be hard to reach (10, 11). The new IHC model has the potential to change the way healthcare is delivered in Italy drastically. Telemedicine can assist in providing care at the patient's home, increase access to healthcare services, and improve patients' health through continuous and dedicated care delivery.

The implementation of this IHC new digital model, in fact, is aimed at strengthening the territorial primary care, in line with the principle of proximity of care: it can reduce waiting times and prevent patient health conditions from worsening by guaranteeing prompt access to the healthcare system, in the form of patient's teleconsultation (*televisita)*, healthcare professionals' teleconsultation, or similar (12). Given the increase in chronic morbidity prevalence, addressing these conditions timely can have a positive impact on hospital admissions (13) by reducing their number and leading to cost optimization and resource savings. This task can be accomplished by the prompt activation of the COT, that can drastically reduce the “near-misses” and the need for excessive follow-up care.

The intent of this new model is to transform the telemedicine as a routine practice. As nearly every doctor could request a teleconsultation from every patient, the opportunity to have a not-in-person visit has been seen to positively impact queuing and waitlists. As seen in other developed countries, such as Australia or the USA, telemedicine adoption leads to shorter waiting lists for specialized outpatient services (14) and the number of waitlisted patients in general (15, 16). Additionally, patients have claimed time savings from less commuting and waiting in queues (17).

Cost-effectiveness of telemedicine should also be considered: available literature on the economics of telemedicine implementation is scarce, but it notes that despite initial set-up expenses could be a significant obstacle to the implementation of telemedicine, they will likely be compensated in the long run (18). Some studies, however, noted that telehealth brings down healthcare system costs in the short- to medium-term, particularly when either patient or clinician traveling was minimized or avoided. Moreover, telemedicine demonstrated enhanced care even when costs were not decreased. For instance, research shows that remote patient monitoring is currently ineffective at cutting costs, but it is good at enhancing general health and lowering morbidity and hospitalization (18). This is in line with the goal of reaching remote areas of the territory, potentially supplying the shortages (both of funds and staff) that primary care has in these areas. Also, these points can help convince the stakeholders to invest and take care of primary care, since very poor efforts have been made toward the latter in recent years (19). In this context, the PNRR has allocated 4 billion euros to strengthen IHC and telemedicine implementation in Italy (20), and it has been estimated there will be a total of 1.2 billion euros in savings due to the new plan's implementation (21).

### Barriers

Some drawbacks should be pointed out in this context.

First, even if conspicuous investments have been made, the Italian technical infrastructural capacity is still very poor, particularly in the areas where telemedicine is needed the most: only around 18% of rural households are reached by Very High Capacity Networks (22). Poor network coverage can limit access to essential services, including emergency services, telemedicine, and online education. This can be especially critical in times of crisis or when remote services are needed.

Furthermore, it should be mentioned that rural systems and older adult population are typically reluctant to change, making the process more difficult (11). In some nations, the lack of computer or digital health literacy was identified as the biggest obstacle to the use of telemedicine (23, 24), so it is possible to expect similar problems in Italy, as it is still below the mean European threshold for digital skills (22). So, if it is evident that adequate infrastructure and sufficient digital literacy is needed to carry out a *televisita* or a teleconsultation, it is worth mentioning that both doctors and patients must receive an adequate training: according to the latest Report of the Digital Health Innovation Observatory, only 60% of primary care physicians have basic digital skills and only about 38% of the Italian population had heard about the EHR, so there's still a long way to go (25).

Additionally, the Italian NHS is highly fragmented at regional level (26), so different outcomes stemming from different resources could be expected. Regions with the highest funding may invest more, not only on digital tools and technical support, but also on infrastructure: broadband connection, updated operative systems, latest hardware, etc (27). Italy is a diverse country, and each region has its unique strengths and challenges. However, there are regions that face specific socio-economic difficulties and are often considered less prosperous in certain respects (for example, southern regions of Italy). It will be important to support less virtuous regions, in order to guarantee the principle of equity of care, and to avoid a “health journey” (the migration of people, usually from southern regions to northern regions, seeking for better healthcare standards).

Finally, it is worth mentioning that Italy is facing a severe challenge related to the shortage of doctors and healthcare professionals. The implementation and sustenance of this new digitally based healthcare model will require the support of trained and skilled medical and technical personnel. If future health care planning will not include a plan for recruitment and adequate professional education, it will be complicated to govern the vast change that the new model will cause on a regional and national scale.

## Future implications

In 1995, Pisanelli et al. predicted that telemedicine would play a major role in Italy, improving the quality of health care and reducing costs (28). More than 20 years later, despite the epidemic input, records show that telemedicine is still only seldomly used (29). Italy is still far behind other nations where COVID has made a strong push (as in the US) (30), but this gap can be addressed by improving acceptance by end-users (31, 32). For this reason, it is important that health professionals, the media, and institutions promote clear, accurate and transparent communication and information on the new model, to involve the population and let them know the benefit of this approach.

Another aspect to consider is the role of COT and the introduction of the EHR. Compared to the current situation, it will be possible to see greater communication, not only between the territory and the patient, but also between the territory and the Community Health Houses, or between the territory and the hospital structure belonging to that district. All of this can happen both with the introduction of a new organization responsible for coordinating these elements (the COT), and with the unification of health data with the EHR. The possibility of having such uniformity will make both accessibility and decision making easier and faster in the transfer of patients between one structure and another, as well as in acute and chronic management, always in an integrated vision.

It is important to address the challenge posed by the “inverse care law”: people who need health assistance the most, will be the ones to have bigger difficulties to access digital healthcare or engage with digital platforms, e.g., disadvantaged groups suffering from poverty, language diversity, or disability. A crucial point is age: some studies found that telemedicine use was decreased as people aged (33, 34). Since 34.9% of the Italian population will be 65 or older by 2050 (9), policymakers and healthcare professionals will need to address the technology gap in older adult patients when implementing telemedicine and telehealth. When it comes to consulting a medical report, engaging in a patient's teleconsultation (*televisita*), or employing a telemonitoring smart bracelet, education and communication is the key to make things run smoothly. Formal programs and updated courses will be needed to embrace the digital illiterate and to prevent the already present gap from expanding further.

That said, strengthening communication of the benefits that telemedicine can bring to healthcare will be crucial to avoid frustration and ensure a smooth adoption by end-users (35). Policymakers and other stakeholders should address factors that might affect implementation, such as fostering teamwork and involving and supporting frontline employees, in order to ensure a successful application (36). In this sense, the Italian pace has been slow, but it is important to run toward the equality of access and the full integration at the national level of telemedicine services (37).

Although various tools and technologies are used for the provision of digitally integrated home care services within the various European countries, it is difficult to identify a nationally approved operational plan which regulates the use of remote home care technologies in a practical and definitive manner, such as the one just published in Italy.

One of the countries at a global level that has shown great commitment to the development of a digital ecosystem in the field of home care assistance is Australia. The government has included a specific plan on this area in its national agenda (“Australia's National Digital Health Strategy”) that implements digitally enabled models of care. The successful implementation of this Strategy is contingent on overcoming challenges related to data security, privacy, interoperability, and the engagement of both healthcare professionals and patients. Nevertheless, it also has the potential to improve the patient care, to enhance efficiency (administrative processes, reduce paperwork, and minimize redundant tests and procedures), to guarantee a better access to health records, to increase interoperability of the health system, and to provide telehealth and remote monitoring.

## Author contributions

FC: Investigation, Supervision, Visualization, Writing—original draft, Writing—review & editing. AG: Investigation, Methodology, Writing—original draft, Writing—review & editing. AM: Investigation, Writing—original draft. FB: Investigation, Writing—original draft. FAC: Methodology, Writing—review & editing. VS: Writing—original draft. SB: Conceptualization, Writing—review & editing. SP: Writing—review & editing. AB: Supervision, Writing—review & editing. MB: Writing—review & editing. EC: Writing—original draft. WR: Visualization, Writing—review & editing.
